# Supplementary material for: Shared decision making interventions in mental healthcare: a protocol for an umbrella review
Source: BMJ Open. 2021 Sep 15;11(9):e051283. doi: 10.1136/bmjopen-2021-051283 (PMC8444255; doi:10.1136/bmjopen-2021-051283)
Supplement: Author's manuscript [file bmjopen-2021-051283.draft_revisions.pdf]

BMJ Open is committed to open peer review. As part of this commitment we make the peer review history of every article we publish publicly available.

When an article is published we post the peer reviewers' comments and the authors' responses online. We also post the versions of the paper that were used during peer review. These are the versions that the peer review comments apply to.

The versions of the paper that follow are the versions that were submitted during the peer review process. They are not the versions of record or the final published versions. They should not be cited or distributed as the published version of this manuscript.

BMJ Open is an open access journal and the full, final, typeset and author-corrected version of record of the manuscript is available on our site with no access controls, subscription charges or pay-per-view fees (http://bmjopen.bmj.com).

If you have any questions on BMJ Open's open peer review process please email info.bmjopen@bmj.com

BMJ Open

Shared decision making interventions in mental health care: A protocol for an umbrella review

Journal:	*BMJ Open*
Manuscript ID	bmjopen-2021-051283
Article Type:	Protocol
Date Submitted by the Author:	14-Mar-2021
Complete List of Authors:	Chmielowska, Marta; University College London Faculty of Brain Sciences, Clinical, Educational and Health Psychology; North East London NHS Foundation Trust Goodmayes Hospital, Research and Development<br>Zisman-Ilani, Yaara; Temple University, Department of Rehabilitation Sciences, College of Public Health<br>Saunders, Rob ; University College London Faculty of Brain Sciences, Clinical, Educational and Health Psychology<br>Pilling, Stephen; University College London Faculty of Brain Sciences, Clinical, Educational and Health Psychology
Keywords:	Protocols & guidelines < HEALTH SERVICES ADMINISTRATION & MANAGEMENT, MENTAL HEALTH, PSYCHIATRY

SCHOLARONE™
Manuscripts

I, the Submitting Author has the right to grant and does grant on behalf of all authors of the Work (as defined in the below author licence), an exclusive licence and/or a non-exclusive licence for contributions from authors who are: i) UK Crown employees; ii) where BMJ has agreed a CC-BY licence shall apply, and/or iii) in accordance with the terms applicable for US Federal Government officers or employees acting as part of their official duties; on a worldwide, perpetual, irrevocable, royalty-free basis to BMJ Publishing Group Ltd ("BMJ") its licensees and where the relevant Journal is co-owned by BMJ to the co-owners of the Journal, to publish the Work in this journal and any other BMJ products and to exploit all rights, as set out in our licence.

The Submitting Author accepts and understands that any supply made under these terms is made by BMJ to the Submitting Author unless you are acting as an employee on behalf of your employer or a postgraduate student of an affiliated institution which is paying any applicable article publishing charge ("APC") for Open Access articles. Where the Submitting Author wishes to make the Work available on an Open Access basis (and intends to pay the relevant APC), the terms of reuse of such Open Access shall be governed by a Creative Commons licence – details of these licences and which Creative Commons licence will apply to this Work are set out in our licence referred to above.

Other than as permitted in any relevant BMJ Author's Self Archiving Policies, I confirm this Work has not been accepted for publication elsewhere, is not being considered for publication elsewhere and does not duplicate material already published. I confirm all authors consent to publication of this Work and authorise the granting of this licence.

For peer review only

Shared decision making interventions in mental health care: A protocol for an umbrella review

Authors: Marta Chmielowska[1,2], Yaara Zisman-Ilani[3], Rob Saunders[1], Steve Pilling[1]

Affiliations:

1. Research Department of Clinical, Educational and Health Psychology, University College London (UCL), London, UK.
2. The North East London NHS Foundation Trust (NELFT) Research and Development Department, London, UK.
3. Social and Behavioral Sciences, Temple University College of Public Health, Philadelphia, Pennsylvania, USA.

Email Addresses: MC: m.chmielowska@ucl.ac.uk; YZ: yaara@temple.edu; RS: r.saunders@ucl.ac.uk; SP: s.pilling@ucl.ac.uk

Corresponding Author: Marta Chmielowska, m.chmielowska@ucl.ac.uk Research Department of Clinical, Educational and Health Psychology, University College London (UCL), London, UK.

Abstract word count = 296; **Main text word count =** 2362.

For peer review only - http://bmjopen.bmj.com/site/about/guidelines.xhtml

ABSTRACT

Introduction: Shared decision making (SDM) has been advocated as a key component of person-centred care and recovery from mental illness. Although the principles of SDM have been well documented, there is a lack of guidance about how to accomplish SDM in mental health care. The objective of the present protocol is to describe the methods for an umbrella review to determine the effectiveness elements of SDM interventions for persons diagnosed with a mental illness. An umbrella review's key characteristic is that it only considers for inclusion the highest level of evidence, namely other systematic reviews and meta-analyses.

Methods and Analysis: Electronic searches will be performed in CINAHL, PubMed, Scopus, Ovid MEDLINE, Ovid EMBASE, Ovid Cochrane Library, Web of Science, Scopus and Ovid PsycInfo. Based on Joanna Briggs Institute recommended guidelines, review articles will be included if they were published between 2010 and 2021. This approach will help identify current and emerging evidence-based treatment options in mental illness. Included articles will be assessed for quality using AMSTAR 2 tool and ratings of the quality of evidence in each review. Presentation of results will align with guidelines in the Cochrane Handbook for Systematic Reviews of Interventions and the Preferred Reporting Items for Systematic Reviews and Meta-Analyses (PRISMA) 2020 statement. Findings will be stratified by mode of intervention and implementation characteristics and will inform development of SDM taxonomy in mental healthcare.

Ethics and dissemination: This umbrella review will focus on the analysis of secondary data and does not require ethics approval. Findings will be disseminated widely to clinicians, researchers, and services users via journal publication, conference presentations, and social media. The results will contribute to the conceptualization and understanding of effective SDM interventions in mental health care and to improving the quality of SDM for individuals with a mental illness.

For peer review only - http://bmjopen.bmj.com/site/about/guidelines.xhtml

ARTICLE SUMMARY

Strengths and limitations of this study

- This will be the first umbrella review of systematic review articles about SDM in mental health.
- This approach will allow for a comprehensive review of a very broad topic by summarising the evidence from multiple research syntheses into one systematic review of reviews.
- Findings will provide the needed evidence for effective SDM elements in mental healthcare and point to the needed methodological improvements and existing gaps.
- It will be a critical first step towards developing a taxonomic classification of SDM in mental healthcare.
- The search will be restricted to English and might exclude additional studies published in other languages.

Keywords
Umbrella review, Overview of systematic reviews, Review of reviews, Shared decision-making interventions, Mental illness

For peer review only

For peer review only - http://bmjopen.bmj.com/site/about/guidelines.xhtml

INTRODUCTION

Shared decision making (SDM) is a health communication approach that focuses on improving patient–clinician interactions around medical decisions in chronic conditions with the ultimate goal of improving clinical and functional outcomes.[1–4] Overall benefits of SDM are well established, including reduced decisional conflict, increased knowledge, satisfaction with care, participation in decision-making, greater treatment engagement, and improved clinical outcomes.[5–6] In the last 15 years, SDM has been advocated as the recommended model for treatment and rehabilitation decision-making among people affected by mental illness, given that self-determination, choice, and autonomy, core principals of an SDM process, are also core aspects of recovery-oriented care. [7-13] Yet, rates of SDM implementation and use in mental health are still very low compared to physical healthcare.[14-16] The literature on SDM in mental illness draws attention to several barriers to SDM implementation, including prevailing stigma among patients and clinicians regarding the patient ability and capacity to make decisions, and issues related to clinicians' fear of liability and legal exposure. [11,13–16]

Another important barrier to SDM promotion in mental health care is the lack of clear definition of SDM practice and the limited understanding of what are the key components of effective SDM interventions in mental health.[10] Currently, and uniquely to mental illness, SDM is interpreted using a wide range of definitions and different types of SDM interventions and practices, which cause confusion and make it hard to standardize SDM as part of a routine mental health practice. Therefore, there is a need to define what is considered an effective SDM approach in mental health care and to determine the core elements and steps which are required for its successful implementation in mental health populations. It may be especially the case in situations where the possibility of involuntary hospitalization creates extreme forms of 'power asymmetry' and where the importance of long-term adherence requires special attention for patient satisfaction with their treatment.[13]

This protocol describes the methods for an upcoming umbrella review to identify and define the effectiveness elements of SDM interventions in mental health care, and to support the implementation of SDM principles in clinical practice.

For peer review only - http://bmjopen.bmj.com/site/about/guidelines.xhtml

OBJECTIVES

1. Identify all the recently published systematic reviews and/or meta-analyses which report on the effectiveness of SDM interventions for care and/or treatment of mental health disorders.
2. Assess the scope, and quality of the identified systematic review articles and to provide a more comprehensive account of the available evidence for the effectiveness of SDM, including key components or principles associated with better outcomes.
3. Develop a taxonomic classification of SDM in mental healthcare which will be used as a guide for implementation of evidence-based interventions for care and treatment of mental disorders.

For peer review only

For peer review only - http://bmjopen.bmj.com/site/about/guidelines.xhtml

METHODS

Protocol and registration

Methods for this umbrella review were developed using criteria for conducting overviews of reviews in the Cochrane Handbook of Systematic Reviews of Interventions. This protocol is registered on the International prospective register of systematic reviews (PROSPERO: CRD42020190700). Only published studies will be examined for this review and no ethical approval is required.

Patient and Public Involvement

Patients and/or the public will not be directly involved in this study.

Eligibility criteria

The reviews considered to be systematic will be included if the authors defined a strategy to search for studies, to appraise their quality and to synthesise their findings. These may consist of reviews of randomised trials, non-randomised trials, and before-and-after studies (BAs). The excluded articles for the current review will consist of non-systematic reviews and studies that involve primary data collection including but are not limited to, randomised trials and non-randomised trials. The main focus will be on systematic reviews rather than original trials in order to utilise the widest range of relevant evidence and compare the best estimates of effectiveness of different interventions. Reviews will be included regardless of the statistical significance of the reported results. In a situation where the same group of authors published more than one systematic review of the same intervention and patient population, the most recent review will be selected if considered by its authors as an update of their previous review(s). If two or more reviews of the same intervention and patient population are published in a short period of time (<2 years) but with conflicting results, any potential similarities and/or differences will be explored in the full texts of the reviews and lists of included studies. The comparison results will be tabulated, including the rationale for the selection of reviews.

Quality criteria

To ensure the identified reviews are 'systematic' they would be required to meet the minimum level of methodological rigour, and include studies which addressed the following two items of the Assessment of Multiple Systematic Reviews (AMSTAR) 2 tool[17]: *Did the review authors use a comprehensive literature search strategy (e.g. were at least two databases searched)?* and *Did the review authors use a satisfactory technique for assessing the risk of bias (RoB) in individual studies that were included in the review (e.g. allocation)?* Other umbrella review authors have used similar criteria[18] or limited inclusion to only Cochrane reviews to ensure a

For peer review only - http://bmjopen.bmj.com/site/about/guidelines.xhtml

minimum level of quality and rigour.[19-20] Therefore, this approach will enhance acceptability and feasibility of the proposed umbrella review.

Types of interventions

The included reviews may consist of studies where interventions were provided by a wide range of healthcare professionals. Interventions could target patients (e.g., patient-mediated interventions), healthcare professionals (e.g., distribution of printed educational material); or both (e.g., a patient-mediated intervention combined with an intervention targeting healthcare professionals). Interventions could take place in any setting (e.g., inpatient, outpatient, primary care, community, secure environment) and will not be restricted by the mode or intensity of delivery. This protocol will rely on the NICE working definition of SDM which is referred to as a collaborative process through which a healthcare professional supports a person to reach a joint decision about their care.[21] The included reviews may consist of studies which compared SDM interventions to other interventions with a similar purpose, or with usual care.

Types of participants

Participants will include adults (aged 18 years and over) who have been diagnosed with a mental health disorder and are facing a decision about their mental health treatment. A mental health disorder will be defined as diagnosable psychological problems which can disrupt thinking, feeling, moods and behaviours, and can cause significant impairment in one's day-to-day functioning. Examples are mood disorders, anxiety disorders, personality disorders, eating disorders, alcohol and substance use disorders, schizophrenia and psychotic disorders. The excluded systematic reviews will target populations other than adults as well as patients diagnosed with Mild Cognitive Impairment, Dementia, Learning Disabilities, and an Acquired Brain Injury.

Outcome measures

The identified SDM practices will be assessed with one of the following types of measures: observer measures, professional-report, and/or patient-report tools.[22] A wide range of decision outcomes will be reported and summarised to provide a greater insight into the decision-making process. As proposed by Kreps and colleagues in their Transformation Model of Communication and Health Outcomes[23], patient outcomes will be classified by their impact on the individual across three categories: affective-cognitive, behavioural, and physiological. Affective-cognitive outcomes include knowledge, attitudinal, and affective/ emotional effects. Behavioural outcomes include adherence to recommended treatments and adoption of health behaviours. Physiological outcomes include measures quality of life, self-rated health, and

For peer review only - http://bmjopen.bmj.com/site/about/guidelines.xhtml

biological measures of health.[23] The reviews which extracted all measures of SDM and all mental health outcomes from eligible studies will be included, regardless of the type of outcome measure used or whether the measurement is subjective or objective in nature.

Search strategy for identification of relevant studies

Nine databases will be searched: CINAHL, PubMed, Scopus, Ovid MEDLINE, Ovid EMBASE, Ovid Cochrane Library, Web of Science, Scopus and Ovid PsycInfo. References will be managed using Endnote X9. The included systematic reviews will be published between 2010 and 2021 and will be limited to English language only. Reviews published before 2010 will be excluded (as per Joanna Briggs Institute guidance), because those published in the past 10 years are considered to represent the contemporary evidence base and will capture primary research conducted over the previous 30 or so years.[24] All the drafted and applied search strategies will not be publicly available until the review is complete.

Selection of studies

The initial screen of titles and abstracts will be performed by the primary researcher, with a random 10% sample screened by a second reviewer. Disagreements will be resolved by discussion between the reviewers, with a senior reviewer acting as arbiter where necessary. Full text screening of potentially relevant studies will then be performed.

Data extraction and management

Data will be extracted independently by two reviewers using a previously designed data extraction form. Discrepancies will be resolved by consensus. Where agreement cannot be reached a senior reviewer will consider the paper and a majority decision will be reached. The data extraction form will include the following details where relevant to the study design: an assessment of methodological quality of the included review; the objectives of the review; a summary of the included studies; the interventions studied, the control conditions (if appropriate); the outcomes and time-points assessed/evaluated and where relevant estimates of effectiveness, and precision; an assessment of the methodological quality and/or risk of bias of the included trials and judgements of the quality of the body of evidence. This information will be valuable in order to map the existing evidence. It will also be necessary to identify potential discrepancies in the result of similar reviews.

Assessment of methodological quality of included reviews

The AMSTAR 2 tool[17] will be used to assess methodological quality of systematic reviews that include both randomized and non-randomized studies of healthcare interventions. The tool

For peer review only - http://bmjopen.bmj.com/site/about/guidelines.xhtml

provides guidance to rate the overall confidence in the results of a review (high, moderate, low or critically low depending on the number of critical flaws and/or non-critical weaknesses). Given that this is an updated version of AMSTAR, this tool will be preferred for use in future umbrella reviews/overviews. The quality appraisal will include: a table that provides a breakdown of how each systematic review was rated on each question of the tool, the rationale behind the assessments, and an overall rating for each systematic review. The results of the quality/risk of bias assessments will be then used to help contextualize the umbrella review's evidence base (e.g., by assessing whether and to what extent SR methods may have affected the umbrella review's comprehensiveness and results). Two umbrella review authors will assess the quality of each individual text. Discrepancies will be resolved by consensus.

Assessment of the quality of the evidence in reviews

The Grading of Recommendations Assessment, Development and Evaluation (GRADE) ratings will be extracted from each included review. This approach provides guidance on rating the quality of research evidence in health care and has been widely implemented by organisations such as the World Health Organization, Cochrane Collaboration, Agency for Healthcare Research and Quality (USA) and National Institute of Health and Care Excellence (UK). Similar to previous umbrella reviews/overviews, the authors will make judgments to downgrade or upgrade the quality of evidence based on the risk of bias using criteria specified by the GRADE working group. Discrepancies in the ratings of the quality of evidence will be resolved by consensus between the authors and, if necessary, arbitration by a senior reviewer.

Data synthesis and presentation

A rigorous international gold-standard methodology of the Preferred Reporting Items for Systematic Reviews and Meta-Analyses (PRISMA) 2020[25] will be employed to facilitate the development and reporting of this protocol. PRISMA 2020 guideline will improve the transparency, accuracy and completeness of the umbrella review protocol. It is expected that the articles will vary considerably both in terms of their review methodology and reporting of outcomes. The presented comparisons will be primarily determined by the content of the included reviews. Data will be grouped where possible according to the population, the type of intervention, and outcome measure. Important limitations within the evidence base will be presented and discussed. Any possible influence of publication/small study biases on review findings will be also considered. Finally, a list of recommendations based on the data synthesis from all studies will be compiled.

Subgroup analysis

For peer review only - http://bmjopen.bmj.com/site/about/guidelines.xhtml

Within the SDM literature, a distinction will be made between interventions targeting patients, interventions targeting healthcare professionals and interventions targeting both. This umbrella review will examine the specific nature of SDM interventions used in each context and evaluate if particular types of intervention are more effective for treatment of mental health disorders.

For peer review only

For peer review only - http://bmjopen.bmj.com/site/about/guidelines.xhtml

DISCUSSION

Despite widespread support for involving patients with mental illness in decisions about their care, SDM is not yet the norm. Inconsistent definitions and measurement of SDM can complicate efforts to identify the relationships between SDM and patient reported outcomes and to make any meaningful comparisons across studies. The available evidence for the effectiveness of SDM interventions in mental health care is inconclusive when compared to the evidence from the other fields of medicine such as diabetes or cancer. The present protocol describes the methods and steps for an umbrella review of systematic reviews and meta-analyses of SDM interventions for persons diagnosed with a mental illness. By facilitating conceptual and practical developments, the review will narrow the current gap between theoretical and policy ideals, and clinical realities in an important area of mental health practice. If the results of this umbrella review are translated into changes in patient care and healthcare practices, then patients will benefit from the reduced burden of hospitalisation either through improved disease treatment and management or better preventative care.

Competing interests

None declared

For peer review only

REFERENCES

1. Charles C, Gafni A, Whelan T. Shared decision-making in the medical encounter: what does it mean?(or it takes at least two to tango). *Soc Sci Med*. 1997;44(5):681-692.
2. Charles C, Gafni A, Whelan T. Decision-making in the physician–patient encounter: revisiting the shared treatment decision-making model. *Soc Sci Med*. 1999;49(5):651-661.
3. Elwyn G, Frosch D, Thomson R, et al. Shared decision making: a model for clinical practice. *J Gen Intern Med*. 2012;27(10):1361-1367. doi:10.1007/s11606-012-2077-6
4. Barry MJ, Edgman-Levitan S. Shared decision making—the pinnacle of patient-centered care. *N Engl J Med*. 2012;366(9):780-781.
5. Deegan PE, Drake RE. Shared decision making and medication management in the recovery process. *Psychiatr Serv*. 2006;57(11):1636.
6. Drake RE, Deegan PE, Rapp C. The promise of shared decision making in mental health. *Psychiatr Rehabil J*. 2010;34(1):7-13.
7. Slade M, Amering M, Farkas M, et al. Uses and abuses of recovery: implementing recovery-oriented practices in mental health systems. *World Psychiatry*. 2014;13(1):12-20. doi:10.1002/wps.20084
8. Zisman-Ilani Y, Barnett E, Harik J, et al. O'Connell M. Expanding the concept of shared decision making for mental health: A systematic and scoping review of interventions. *Ment Heal Rev J*. 2017;22(3):191-213. doi:10.1108/MHRJ-01-2017-0002
9. Zisman-Ilani Y, Roe D, Elwyn G, et al. Shared Decision Making for Psychiatric Rehabilitation Services Before Discharge from Psychiatric Hospitals. *Health Commun*. 2018. doi:10.1080/10410236.2018.1431018
10. Hamann J, Heres S. Adapting Shared Decision Making for Individuals With Severe Mental Illness. *Psychiatr Serv*. 2014;65(12):1483-1486.
11. Zisman-Ilani Y, Lysaker P, Hasson-Ohayon I. Shared risk-taking: shared decision making in serious mental illness. *Psychiatr Serv*. 2021.
12. Drake RE, Deegan PE. Shared decision making is an ethical imperative. *Psychiatr Serv*. 2009;60(8):1007.
13. Hamann J, Mendel R, Cohen R, et al. Psychiatrists' use of shared decision making in the treatment of schizophrenia: patient characteristics and decision topics. *Psychiatr Serv*. 2009;60(8):1107-1112.
14. Hamann J, Kolbe G, Leucht S. Medical decision making in the mental health field. *Psychiatr Serv*. 2005;56(2):221. doi:https://dx.doi.org/10.1176/appi.ps.56.2.221
15. Hamann J, Bühner M, Rüsch N. Self-Stigma and Consumer Participation in Shared Decision Making in Mental Health Services. *Psychiatr Serv*. 2017;68(8):783-788.

For peer review only

doi:10.1176/appi.ps.201600282

16. Zisman-Ilani Y, Hasson-Ohayon I, Levy-Frank I, et al. Self-stigma, insight, and family burden among Israeli mothers of people with serious mental illness: Ethno-national considerations. *Transcult Psychiatry*. 2017;54(3). doi:10.1177/1363461517703022
17. Shea BJ, Reeves BC, Wells G, et al. AMSTAR 2: a critical appraisal tool for systematic reviews that include randomised or non-randomised studies of healthcare interventions, or both. *BMJ.* 2017;358:j4008. doi: 10.1136/bmj.j4008.
18. Mickan S, Tilson JK, Atherton H, et al. Evidence of effectiveness of health care professionals using handheld computers: a scoping review of systematic reviews. *J Med Internet Res.* 2013;15:e212. doi: 10.2196/jmir.2530.
19. Nunan D, Mahtani KR, Roberts N, et al. Physical activity for the prevention and treatment of major chronic disease: an overview of systematic reviews. *Syst Reviews.* 2013;2:56 doi: 10.1186/2046-4053-2-56.
20. van der Wouden JC, Bueving HJ, Poole P. Preventing influenza: an overview of systematic reviews. *Respiratory Medicine.* 2005;99:1341–1349.
21. NICE Advice. Shared decision making. 2019. https://www.nice.org.uk/advice/ktt23/resources/shared-decision-making-pdf-58758011521477 (Accessed on 7th March 2021).
22. Scholl I, Koelewijn-van Loon M, Sepucha K, et al. Measurement of shared decision making–a review of instruments. *Z Evid Fortbild Qual Gesundhwes.* 2011 1;105(4):313-324. doi: 10.1016/j.zefq.2011.04.012.
23. Kreps GL, O'Hair DA, Clowers M. The influences of human communication on health outcomes. *Am Behav Sci.* 1994;38(2):248-256.
24. Aromataris E, Fernandez R, Godfrey CM, Holly C, Khalil H, Tungpunkom P. Summarizing systematic reviews: methodological development, conduct and reporting of an umbrella review approach. *Int J Evid Based Healthc.* 2015;13(3):132–40. doi: 10.1097/XEB.0000000000000055.
25. Page MJ, McKenzie J, Bossuyt P. The PRISMA 2020 statement: an updated guideline for reporting systematic reviews. 2020. doi:10.31222/osf.io/v7gm2

For peer review only - http://bmjopen.bmj.com/site/about/guidelines.xhtml

FOOTNOTES

Acknowledgements

The protocol of the current umbrella review was registered in the PROSPERO database: CRD42020190700.

Authors' contributions

MC wrote the first draft of the protocol. YZI and RS read and revised the draft further. All authors approved the final version of the manuscript and are accountable for all aspects of the work.

Funding

This work was supported by the National Institute for Health Research (NIHR), HSDR Project Number: RP-PG-0615-2002.

Availability of data and materials

The datasets used and/or analysed during the umbrella review will be available from the corresponding author on reasonable request.

Competing interests

None declared.

Word Count

2,362 words.

For peer review only

Reporting checklist for protocol of a systematic review and meta analysis.

Based on the PRISMA-P guidelines.

Instructions to authors

Complete this checklist by entering the page numbers from your manuscript where readers will find each of the items listed below.

Your article may not currently address all the items on the checklist. Please modify your text to include the missing information. If you are certain that an item does not apply, please write "n/a" and provide a short explanation.

Upload your completed checklist as an extra file when you submit to a journal.

In your methods section, say that you used the PRISMA-Preporting guidelines, and cite them as:

Moher D, Shamseer L, Clarke M, Ghersi D, Liberati A, Petticrew M, Shekelle P, Stewart LA. Preferred Reporting Items for Systematic Review and Meta-Analysis Protocols (PRISMA-P) 2015 statement. Syst Rev. 2015;4(1):1.

		Reporting Item	Page Number
**Title**			
Identification	#1a	Identify the report as a protocol of a systematic review	1, 2, 4
Update	#1b	If the protocol is for an update of a previous systematic review, identify as such	N/A
**Registration**			
	#2	If registered, provide the name of the registry (such as PROSPERO) and registration number	6, 14
**Authors**			
Contact	#3a	Provide name, institutional affiliation, e-mail address of all protocol authors; provide physical mailing address of corresponding author	1
Contribution	#3b	Describe contributions of protocol authors and identify the guarantor of the review	14

For peer review only

**Amendments**			
	#4	If the protocol represents an amendment of a previously completed or published protocol, identify as such and list changes; otherwise, state plan for documenting important protocol amendments	N/A
**Support**			
Sources	#5a	Indicate sources of financial or other support for the review	14
Sponsor	#5b	Provide name for the review funder and / or sponsor	14
Role of sponsor or funder	#5c	Describe roles of funder(s), sponsor(s), and / or institution(s), if any, in developing the protocol	N/A
**Introduction**			
Rationale	#6	Describe the rationale for the review in the context of what is already known	2, 4
Objectives	#7	Provide an explicit statement of the question(s) the review will address with reference to participants, interventions, comparators, and outcomes (PICO)	4, 5
**Methods**			
Eligibility criteria	#8	Specify the study characteristics (such as PICO, study design, setting, time frame) and report characteristics (such as years considered, language, publication status) to be used as criteria for eligibility for the review	6, 7, 8
Information sources	#9	Describe all intended information sources (such as electronic databases, contact with study authors, trial registers or other grey literature sources) with planned dates of coverage	8
Search strategy	#10	Present draft of search strategy to be used for at least one electronic database, including planned limits, such that it could be repeated	N/A
Study records - data management	#11a	Describe the mechanism(s) that will be used to manage records and data throughout the review	8
Study records - selection process	#11b	State the process that will be used for selecting studies (such as two independent reviewers) through each phase of the review (that is, screening, eligibility and inclusion in meta-analysis)	8

For peer review only

Study records - data collection process	#11c	Describe planned method of extracting data from reports (such as piloting forms, done independently, in duplicate), any processes for obtaining and confirming data from investigators	8
Data items	#12	List and define all variables for which data will be sought (such as PICO items, funding sources), any pre-planned data assumptions and simplifications	7
Outcomes and prioritization	#13	List and define all outcomes for which data will be sought, including prioritization of main and additional outcomes, with rationale	7, 8
Risk of bias in individual studies	#14	Describe anticipated methods for assessing risk of bias of individual studies, including whether this will be done at the outcome or study level, or both; state how this information will be used in data synthesis	9
Data synthesis	#15a	Describe criteria under which study data will be quantitatively synthesised	9
Data synthesis	#15b	If data are appropriate for quantitative synthesis, describe planned summary measures, methods of handling data and methods of combining data from studies, including any planned exploration of consistency (such as I2, Kendall's τ)	9
Data synthesis	#15c	Describe any proposed additional analyses (such as sensitivity or subgroup analyses, meta-regression)	9, 10
Data synthesis	#15d	If quantitative synthesis is not appropriate, describe the type of summary planned	N/A
Meta-bias(es)	#16	Specify any planned assessment of meta-bias(es) (such as publication bias across studies, selective reporting within studies)	9
Confidence in cumulative evidence	#17	Describe how the strength of the body of evidence will be assessed (such as GRADE)	9

The PRISMA-P elaboration and explanation paper is distributed under the terms of the Creative Commons Attribution License CC-BY. This checklist was completed on 14. March 2021 using https://www.goodreports.org/, a tool made by the EQUATOR Network in collaboration with Penelope.ai

For peer review only

BMJ Open

Shared decision making interventions in mental health care: A protocol for an umbrella review

Journal:	*BMJ Open*
Manuscript ID	bmjopen-2021-051283.R1
Article Type:	Protocol
Date Submitted by the Author:	25-May-2021
Complete List of Authors:	Chmielowska, Marta; University College London, Centre for Outcomes Research and Effectiveness (CORE), Research Department of Clinical, Educational and Health Psychology; North East London NHS Foundation Trust Goodmayes Hospital, Research and Development<br>Zisman-Ilani, Yaara; Temple University College of Public Health, Social and Behavioral Sciences<br>Saunders, Rob ; University College London, Centre for Outcomes Research and Effectiveness (CORE), Research Department of Clinical, Educational and Health Psychology<br>Pilling, Stephen; University College London, Centre for Outcomes Research and Effectiveness (CORE), Research Department of Clinical, Educational and Health Psychology
**Primary Subject Heading**:	Patient-centred medicine
Secondary Subject Heading:	Mental health, Patient-centred medicine, Communication
Keywords:	Protocols & guidelines < HEALTH SERVICES ADMINISTRATION & MANAGEMENT, MENTAL HEALTH, PSYCHIATRY

SCHOLARONE™
Manuscripts

I, the Submitting Author has the right to grant and does grant on behalf of all authors of the Work (as defined in the below author licence), an exclusive licence and/or a non-exclusive licence for contributions from authors who are: i) UK Crown employees; ii) where BMJ has agreed a CC-BY licence shall apply, and/or iii) in accordance with the terms applicable for US Federal Government officers or employees acting as part of their official duties; on a worldwide, perpetual, irrevocable, royalty-free basis to BMJ Publishing Group Ltd ("BMJ") its licensees and where the relevant Journal is co-owned by BMJ to the co-owners of the Journal, to publish the Work in this journal and any other BMJ products and to exploit all rights, as set out in our licence.

The Submitting Author accepts and understands that any supply made under these terms is made by BMJ to the Submitting Author unless you are acting as an employee on behalf of your employer or a postgraduate student of an affiliated institution which is paying any applicable article publishing charge ("APC") for Open Access articles. Where the Submitting Author wishes to make the Work available on an Open Access basis (and intends to pay the relevant APC), the terms of reuse of such Open Access shall be governed by a Creative Commons licence – details of these licences and which Creative Commons licence will apply to this Work are set out in our licence referred to above.

Other than as permitted in any relevant BMJ Author's Self Archiving Policies, I confirm this Work has not been accepted for publication elsewhere, is not being considered for publication elsewhere and does not duplicate material already published. I confirm all authors consent to publication of this Work and authorise the granting of this licence.

For peer review only

Shared decision making interventions in mental health care: A protocol for an umbrella review

Authors: Marta Chmielowska[1,2], Yaara Zisman-Ilani[3], Rob Saunders[1], Stephen Pilling[1]

Affiliations:

1. Centre for Outcomes Research and Effectiveness (CORE), Research Department of Clinical, Educational and Health Psychology, University College London (UCL), London, UK.
2. The North East London NHS Foundation Trust (NELFT) Research and Development Department, London, UK.
3. Social and Behavioral Sciences, Temple University College of Public Health, Philadelphia, Pennsylvania, USA.

Email Addresses: MC: m.chmielowska@ucl.ac.uk; YZ: yaara@temple.edu; RS: r.saunders@ucl.ac.uk; SP: s.pilling@ucl.ac.uk

Corresponding Author: Marta Chmielowska, m.chmielowska@ucl.ac.uk.
Centre for Outcomes Research and Effectiveness (CORE), Research Department of Clinical, Educational and Health Psychology, University College London (UCL), London, UK.

Abstract word count = 297; **Main text word count =** 2,548.

For peer review only

For peer review only - http://bmjopen.bmj.com/site/about/guidelines.xhtml

ABSTRACT

Introduction: Shared decision making (SDM) has been advocated as a key component of person-centred care and recovery from mental illness. Although the principles of SDM have been well documented, there is a lack of guidance about how to accomplish SDM in mental health care. The objective of the present protocol is to describe the methods for an umbrella review to determine the effectiveness elements of SDM interventions for persons diagnosed with a mental illness. An umbrella review's key characteristic is that it only considers for inclusion the highest level of evidence, namely other systematic reviews and meta-analyses.

Methods and Analysis: Electronic searches will be performed in CINAHL, PubMed, Scopus, Ovid MEDLINE, Ovid EMBASE, Ovid Cochrane Library, Web of Science, Scopus and Ovid PsycInfo. Based on Joanna Briggs Institute recommended guidelines, review articles will be included if they were published between 2010 and 2021. This approach will help identify current and emerging evidence-based treatment options in mental illness. Included articles will be assessed for quality using AMSTAR 2 tool and ratings of the quality of evidence in each review. Presentation of results will align with guidelines in the Cochrane Handbook for Systematic Reviews of Interventions and the Preferred Reporting Items for Systematic Reviews and Meta-Analyses (PRISMA) 2020 statement. Findings will be stratified by mode of intervention and implementation characteristics and will inform development of SDM taxonomy in mental healthcare.

Ethics and dissemination: This umbrella review will focus on the analysis of secondary data and does not require ethics approval. Findings will be disseminated widely to clinicians, researchers, and services users via journal publication, conference presentations, and social media. The results will contribute to the conceptualization and understanding of effective SDM interventions in mental health care and to improving the quality of SDM for individuals with a mental illness.

For peer review only - http://bmjopen.bmj.com/site/about/guidelines.xhtml

ARTICLE SUMMARY

Strengths and limitations of this study

- This will be the first umbrella review of systematic review articles about SDM in mental health.
- This approach will allow for a comprehensive review of a very broad topic by summarising the evidence from multiple research syntheses into one systematic review of reviews.
- The search will be restricted to English and might exclude additional studies published in other languages.
- AMSTAR 2 will be used to enable more detailed assessment of systematic reviews that include randomised and/or non-randomised studies of SDM interventions.
- Presentation of results will align with guidelines in the Cochrane Handbook for Systematic Reviews of Interventions and PRISMA 2020 statement.

Keywords

Umbrella review, Overview of systematic reviews, Review of reviews, Shared decision-making interventions, Mental illness

For peer review only

INTRODUCTION

Shared decision making (SDM) is a health communication approach that focuses on improving patient–clinician interactions around medical decisions in chronic conditions with the ultimate goal of improving clinical and functional outcomes.[1-4] Overall benefits of SDM are well established, including reduced decisional conflict, increased knowledge, satisfaction with care, participation in decision-making, greater treatment engagement, and improved clinical outcomes.[5 6] In the last 15 years, SDM has been advocated as the recommended model for treatment and rehabilitation decision-making among people affected by mental illness, given that self-determination, choice, and autonomy, core principals of an SDM process, are also core aspects of recovery-oriented care.[7-13] Yet, rates of SDM implementation and use in mental health are still very low compared to physical healthcare.[14-16] The literature on SDM in mental illness draws attention to several barriers to SDM implementation, including prevailing stigma among patients and clinicians regarding the patient ability and capacity to make decisions, and issues related to clinicians' fear of liability and legal exposure.[11 13-16]

Another important barrier to SDM promotion in mental health care is the lack of clear definition of SDM practice and the limited understanding of what are the key components of effective SDM interventions in mental health.[10] Currently, and uniquely to mental illness, SDM is interpreted using a wide range of definitions and different types of SDM interventions and practices, which cause confusion and make it hard to standardize SDM as part of a routine mental health practice. Therefore, there is a need to define what is considered an effective SDM approach in mental health care and to determine the core elements and steps which are required for its successful implementation in mental health populations. It may be especially the case in situations where the possibility of involuntary hospitalization creates extreme forms of 'power asymmetry' and where the importance of long-term adherence requires special attention for patient satisfaction with their treatment.[13]

This protocol describes the methods for an upcoming umbrella review to identify and define the effectiveness elements of SDM interventions in mental health care, and to support the implementation of SDM principles in clinical practice.

For peer review only - http://bmjopen.bmj.com/site/about/guidelines.xhtml

OBJECTIVES

1. Identify all the recently published systematic reviews and/or meta-analyses which report on the effectiveness of SDM interventions for care and/or treatment of mental health disorders.
2. Assess the scope, and quality of the identified systematic review articles and to provide a more comprehensive account of the available evidence for the effectiveness of SDM, including key components or principles associated with better outcomes.
3. Develop a taxonomic classification of SDM in mental healthcare which will be used as a guide for implementation of evidence-based interventions for care and treatment of mental disorders.

For peer review only

For peer review only - http://bmjopen.bmj.com/site/about/guidelines.xhtml

METHODS

Protocol and registration

Methods for this umbrella review were developed using criteria for conducting overviews of reviews in the Cochrane Handbook of Systematic Reviews of Interventions. This protocol is registered on the International prospective register of systematic reviews (PROSPERO: CRD42020190700). Only published studies will be examined for this review and no ethical approval is required.

Patient and Public Involvement

Patients and/or the public will not be directly involved in this study.

Eligibility criteria

The reviews considered to be systematic will be included if authors of those reviews defined a strategy to search for studies, to appraise their quality and to synthesise their findings. These may consist of reviews of randomised trials, non-randomised trials, and before-and-after studies (BAs), including qualitative and observational studies, as long as it helps with understanding the variation in outcomes and the mechanism by which the SDM interventions have an impact. The excluded articles for the current review will consist of non-systematic reviews and studies that involve primary data collection including but are not limited to, randomised trials and non-randomised trials. As an umbrella review, the main focus will be on systematic reviews rather than original studies in order to utilise the widest range of relevant evidence and compare the best estimates of effectiveness of different interventions. In a situation where the same group of authors published more than one systematic review of the same intervention and patient population, the most recent review will be selected if considered by its authors as an update of their previous review(s). If two or more reviews of the same intervention and patient population are published in a short period of time (<2 years) but with conflicting results, any potential similarities and/or differences will be explored in the full texts of the reviews and lists of included studies. The comparison results will be tabulated, including the rationale for the selection of reviews.

Quality criteria

To ensure the identified reviews are 'systematic' they would be required to meet the minimum level of methodological rigour, and include studies which addressed the following two items of the Assessment of Multiple Systematic Reviews (AMSTAR) 2 tool[17]: *Did the review authors use a comprehensive literature search strategy (e.g. were at least two databases searched)?* and *Did the review authors use a satisfactory technique for assessing*

For peer review only - http://bmjopen.bmj.com/site/about/guidelines.xhtml

the risk of bias (RoB) in individual studies that were included in the review (e.g. allocation)? Other umbrella review authors have used similar criteria[18] or limited inclusion to only Cochrane reviews to ensure a minimum level of quality and rigour.[19 20] Therefore, this approach will enhance acceptability and feasibility of the proposed umbrella review.

Types of interventions

The included reviews may consist of studies where interventions were provided by a wide range of healthcare professionals. Interventions could target patients (e.g., patient-mediated interventions), healthcare professionals (e.g., distribution of printed educational material); or both (e.g., a patient-mediated intervention combined with an intervention targeting healthcare professionals). They could also target patients' families, carers and caregivers (e.g., family involvement in care planning) or triads of patients, their family members and healthcare professionals (e.g., self-management support). Interventions could take place in any setting (e.g., inpatient, outpatient, primary care, community, secure environment) and will not be restricted by the mode or intensity of delivery. This protocol will rely on the NICE working definition of SDM which is referred to as a collaborative process through which a healthcare professional supports a person to reach a joint decision about their care.[21] Published journal articles on SDM often do not provide a clear definition, or they use a term inconsistently.[22 23] Thus, the aim of this review is to rectify various definitions (and measurements) and develop a taxonomic classification of SDM in mental healthcare. The included reviews may consist of studies which compared SDM interventions to other interventions with a similar purpose, or with usual care.

Types of participants

Participants will include adults (aged 18 years and over) who have been diagnosed with a mental health disorder and are facing a decision about their mental health treatment. A mental health disorder will be defined as diagnosable psychological problems which can disrupt thinking, feeling, moods and behaviours, and can cause significant impairment in one's day-to-day functioning. Examples are mood disorders, anxiety disorders, personality disorders, eating disorders, alcohol and substance use disorders, schizophrenia and psychotic disorders. The excluded systematic reviews will target populations other than adults as well as patients diagnosed with Mild Cognitive Impairment, Dementia, Learning Disabilities, and an Acquired Brain Injury.

Outcome measures

For peer review only - http://bmjopen.bmj.com/site/about/guidelines.xhtml

The identified SDM practices will be assessed with one of the following types of measures: observer measures, professional-report, and/or patient-report tools.[24] A wide range of decision outcomes will be reported and summarised to provide a greater insight into the decision-making process. As proposed by Kreps and colleagues in their Transformation Model of Communication and Health Outcomes,[25] patient outcomes will be classified by their impact on the individual across three categories: affective-cognitive, behavioural, and physiological. Affective-cognitive outcomes include knowledge, attitudinal, and affective/ emotional effects. Behavioural outcomes include adherence to recommended treatments and adoption of health behaviours. Physiological outcomes include measures quality of life, self-rated health, and biological measures of health.[25] The reviews which extracted all measures of SDM and all mental health outcomes from eligible studies will be included, regardless of the type of outcome measure used or whether the measurement is subjective or objective in nature.

Search strategy for identification of relevant studies

Nine databases will be searched: CINAHL, PubMed, Scopus, Ovid MEDLINE, Ovid EMBASE, Ovid Cochrane Library, Web of Science, Scopus and Ovid PsycInfo. References will be managed using Endnote X9. The included systematic reviews will be published between 2010 and 2021 and will be limited to English language only. Reviews published before 2010 will be excluded (as per Joanna Briggs Institute guidance), because those published in the past 10 years are considered to represent the contemporary evidence base and will capture primary research conducted over the previous 30 or so years.[26] The search strategy was initially formulated for Ovid Medline (please see Appendix 1), then further tailored as appropriate for use with other databases. All the drafted and applied search strategies will be publicly available after the review is completed.

Selection of studies

The initial screening of titles and abstracts will be performed by the primary reviewer (MC) with a random 10% sample screened by a second reviewer (YZ). Although a dual-reviewer screening of titles and abstracts is an optimal approach; a single-reviewer screening is an acceptable alternative as stated in the Cochrane Handbook for Systematic Reviews of Interventions.[27] Disagreements will be resolved by discussion between the reviewers, with a senior reviewer acting as arbiter where necessary. Full text screening of potentially relevant studies will then be performed.

Data extraction and management

For peer review only - http://bmjopen.bmj.com/site/about/guidelines.xhtml

Data will be extracted independently by two reviewers using a previously designed data extraction form. Discrepancies will be resolved by consensus. Where agreement cannot be reached a senior reviewer will consider the paper and a majority decision will be reached. The data extraction form will include the following details where relevant to the study design: an assessment of methodological quality of the included review; the objectives of the review; a summary of the included studies; the interventions studied, the control conditions (if appropriate); the outcomes and time-points assessed/evaluated and where relevant estimates of effectiveness, and precision; an assessment of the methodological quality and/or risk of bias of the included trials and judgements of the quality of the body of evidence. This information will be valuable in order to map the existing evidence. It will also be necessary to identify potential discrepancies in the result of similar reviews.

Assessment of methodological quality of included reviews

The AMSTAR 2 tool[17] will be used to assess methodological quality of systematic reviews that include both randomized and non-randomized studies of healthcare interventions. The tool provides guidance to rate the overall confidence in the results of a review (high, moderate, low or critically low depending on the number of critical flaws and/or non-critical weaknesses). Given that this is an updated version of AMSTAR, this tool will be preferred for use in future umbrella reviews/overviews. The quality appraisal will include: a table that provides a breakdown of how each systematic review was rated on each question of the tool, the rationale behind the assessments, and an overall rating for each systematic review. The results of the quality/risk of bias assessments will be then used to help contextualize the umbrella review's evidence base (e.g., by assessing whether and to what extent SR methods may have affected the umbrella review's comprehensiveness and results). Two umbrella review authors will assess the quality of each individual text. Discrepancies will be resolved by consensus.

Assessment of the quality of the evidence in reviews

The Grading of Recommendations Assessment, Development and Evaluation (GRADE) ratings will be extracted from each included review. This approach provides guidance on rating the quality of research evidence in health care and has been widely implemented by organisations such as the World Health Organization, Cochrane Collaboration, Agency for Healthcare Research and Quality (USA) and National Institute of Health and Care Excellence (UK). Similar to previous umbrella reviews/overviews, the authors will make judgments to downgrade or upgrade the quality of evidence based on the risk of bias using criteria specified by the GRADE working group. Discrepancies in the ratings of the quality of

For peer review only - http://bmjopen.bmj.com/site/about/guidelines.xhtml

evidence will be resolved by consensus between the authors and, if necessary, arbitration by a senior reviewer.

Data synthesis and presentation

A rigorous international gold-standard methodology of the Preferred Reporting Items for Systematic Reviews and Meta-Analyses (PRISMA) 2020[28] will be employed to facilitate the development and reporting of this protocol. PRISMA 2020 guideline will improve the transparency, accuracy and completeness of the umbrella review protocol. It is expected that the articles will vary considerably both in terms of their review methodology and reporting of outcomes. The presented comparisons will be primarily determined by the content of the included reviews. Data will be grouped where possible according to the population, the type of intervention, and outcome measure. Barriers and facilitators for implementation will be identified across different articles and collated. Important limitations within the evidence base will be presented and discussed. Any possible influence of publication/small study biases on review findings will be also considered. Finally, a list of recommendations based on the data synthesis from all studies will be compiled.

Subgroup analysis

Within the SDM literature, a distinction will be made between interventions targeting patients; interventions targeting patients' families, carers, and caregivers; interventions targeting healthcare professionals; interventions targeting patients and healthcare professionals; or interventions targeting clinician-patient-family caregiver triads. This umbrella review will examine the specific nature of SDM interventions used in each context and evaluate if particular types of intervention are more effective for treatment of mental health disorders. The ability to conduct statistical analysis will be dependent on the identified reviews, and how data is presented.

For peer review only - http://bmjopen.bmj.com/site/about/guidelines.xhtml

ETHICS AND DISSEMINATION

Despite widespread support for involving patients with mental illness in decisions about their care, SDM is not yet the norm. Inconsistent definitions and measurement of SDM can complicate efforts to identify the relationships between SDM and patient reported outcomes and to make any meaningful comparisons across studies. The available evidence for the effectiveness of SDM interventions in mental health care is inconclusive when compared to the evidence from the other fields of medicine such as diabetes or cancer.

The present protocol describes the methods and steps for an umbrella review of systematic reviews and meta-analyses of SDM interventions for adults over 18 years of age diagnosed with a mental illness. This review will focus on the analysis of secondary data and is exempt from ethics approval. The target audience consists of clinicians, researchers, and service users, who will be reached with tailored materials through journal publications, conference presentations, and social media. By facilitating conceptual and practical developments, the review will narrow the current gap between theoretical and policy ideals, and clinical realities in an important area of mental health practice. If the results of this umbrella review are translated into changes in patient care and healthcare practices, then patients will benefit from the reduced burden of hospitalisation either through improved disease treatment and management or better preventative care.

Authors' contributions

MC wrote the first draft of the protocol. YZI and RS read and revised the draft further. SP served as a driving force behind the concept and provided guidance on how to structure the protocol. All authors approved the final version of the manuscript and are accountable for all aspects of the work.

Competing interests

None declared

Funding

This work was supported by the National Institute for Health Research (NIHR), HSDR Project Number: RP-PG-0615-2002.

For peer review only - http://bmjopen.bmj.com/site/about/guidelines.xhtml

REFERENCES

1. Charles C, Gafni A, Whelan T. Shared decision-making in the medical encounter: what does it mean? (or it takes at least two to tango). *Soc Sci Med* 1997;44(5):681-92. doi: 10.1016/s0277-9536(96)00221-3 [published Online First: 1997/03/01]

2. Charles C, Gafni A, Whelan T. Decision-making in the physician-patient encounter: revisiting the shared treatment decision-making model. *Soc Sci Med* 1999;49(5):651-61. doi: 10.1016/s0277-9536(99)00145-8 [published Online First: 1999/08/19]

3. Elwyn G, Frosch D, Thomson R, et al. Shared decision making: a model for clinical practice. *J Gen Intern Med* 2012;27(10):1361-7. doi: 10.1007/s11606-012-2077-6 [published Online First: 2012/05/24]

4. Barry MJ, Edgman-Levitan S. Shared decision making--pinnacle of patient-centered care. *N Engl J Med* 2012;366(9):780-1. doi: 10.1056/NEJMp1109283 [published Online First: 2012/03/02]

5. Deegan PE, Drake RE. Shared decision making and medication management in the recovery process. *Psychiatric Services* 2006;57(11):1636-39. doi: 10.1176/appi.ps.57.11.1636

6. Drake RE, Deegan PE, Rapp C. The promise of shared decision making in mental health. *Psychiatr Rehabil J* 2010;34(1):7-13. doi: 10.2975/34.1.2010.7.13 [published Online First: 2010/07/10]

7. Slade M, Amering M, Farkas M, et al. Uses and abuses of recovery: implementing recovery-oriented practices in mental health systems. *World Psychiatry* 2014;13(1):12-20. doi: 10.1002/wps.20084 [published Online First: 2014/02/06]

8. Zisman-Ilani Y, Barnett E, Harik J, et al. Expanding the concept of shared decision making for mental health: Systematic search and scoping review of interventions. *Mental Health Review Journal* 2017;22(3):191-213. doi: 10.1108/MHRJ-01-2017-0002

9. Zisman-Ilani Y, Roe D, Elwyn G, et al. Shared Decision Making for Psychiatric Rehabilitation Services Before Discharge from Psychiatric Hospitals. *Health Commun* 2019;34(6):631-37. doi: 10.1080/10410236.2018.1431018 [published Online First: 2018/02/03]

10. Hamann J, Heres S. Adapting shared decision making for individuals with severe mental illness. *Psychiatr Serv* 2014;65(12):1483-6. doi: 10.1176/appi.ps.201400307 [published Online First: 2015/03/11]

11. Zisman-Ilani Y, Lysaker PH, Hasson-Ohayon I. Shared Risk Taking: Shared Decision Making in Serious Mental Illness. *Psychiatr Serv* 2021;72(4):461-63. doi: 10.1176/appi.ps.202000156 [published Online First: 2021/02/10]

For peer review only - http://bmjopen.bmj.com/site/about/guidelines.xhtml

12. Drake RE, Deegan PE. Shared decision making is an ethical imperative. *Psychiatr Serv* 2009;60(8):1007. doi: 10.1176/ps.2009.60.8.1007 [published Online First: 2009/08/04]

13. Hamann J, Mendel R, Cohen R, et al. Psychiatrists' use of shared decision making in the treatment of schizophrenia: patient characteristics and decision topics. *Psychiatr Serv* 2009;60(8):1107-12. doi: 10.1176/ps.2009.60.8.1107 [published Online First: 2009/08/04]

14. Hamann J, Kolbe G, Leucht S. Medical Decision Making in the Mental Health Field. *Psychiatric Services* 2005;56(2):221-21. doi: 10.1176/appi.ps.56.2.221

15. Hamann J, Bühner M, Rüsch N. Self-Stigma and Consumer Participation in Shared Decision Making in Mental Health Services. *Psychiatr Serv* 2017;68(8):783-88. doi: 10.1176/appi.ps.201600282 [published Online First: 2017/04/18]

16. Zisman-Ilani Y, Hasson-Ohayon I, Levy-Frank I, et al. Self-stigma, insight, and family burden among Israeli mothers of people with serious mental illness: Ethno-national considerations. *Transcultural Psychiatry* 2017;54(3):423-41. doi: 10.1177/1363461517703022

17. Shea BJ, Reeves BC, Wells G, et al. AMSTAR 2: a critical appraisal tool for systematic reviews that include randomised or non-randomised studies of healthcare interventions, or both. *BMJ* 2017;358:j4008. doi: 10.1136/bmj.j4008

18. Mickan S, Tilson JK, Atherton H, et al. Evidence of effectiveness of health care professionals using handheld computers: a scoping review of systematic reviews. *J Med Internet Res* 2013;15(10):e212. doi: 10.2196/jmir.2530 [published Online First: 2013/10/30]

19. Nunan D, Mahtani KR, Roberts N, et al. Physical activity for the prevention and treatment of major chronic disease: an overview of systematic reviews. *Syst Rev* 2013;2:56. doi: 10.1186/2046-4053-2-56 [published Online First: 2013/07/11]

20. van der Wouden JC, Bueving HJ, Poole P. Preventing influenza: An overview of systematic reviews. *Respiratory Medicine* 2005;99(11):1341-49. doi: https://doi.org/10.1016/j.rmed.2005.07.001

21. Advice N. Shared decision making https://www.nice.org.uk/advice/ktt23/resources/shared-decision-making-pdf-587580115214772019 [updated 2019. accessed 7th March 2021.

22. Makoul G, Clayman ML. An integrative model of shared decision making in medical encounters. *Patient Educ Couns* 2006;60(3):301-12. doi: 10.1016/j.pec.2005.06.010 [published Online First: 2005/07/30]

For peer review only - http://bmjopen.bmj.com/site/about/guidelines.xhtml

23. Moumjid N, Gafni A, Brémond A, et al. Shared decision making in the medical encounter: are we all talking about the same thing? *Med Decis Making* 2007;27(5):539-46. doi: 10.1177/0272989x07306779 [published Online First: 2007/09/18]

24. Scholl I, Koelewijn-van Loon M, Sepucha K, et al. Measurement of shared decision making - a review of instruments. *Z Evid Fortbild Qual Gesundhwes* 2011;105(4):313-24. doi: 10.1016/j.zefq.2011.04.012 [published Online First: 2011/05/31]

25. Kreps GL, O'Hair D, Clowers M. The influences of human communication on health outcomes. *American Behavioral Scientist* 1994;38:248+.

26. Aromataris E, Fernandez R, Godfrey CM, et al. Summarizing systematic reviews: methodological development, conduct and reporting of an umbrella review approach. *Int J Evid Based Healthc* 2015;13(3):132-40. doi: 10.1097/xeb.0000000000000055 [published Online First: 2015/09/12]

27. Higgins JPTTJ CJ, Cumpston M, Li T, Page MJ, Welch VA, editors. . Cochrane Handbook for Systematic Reviews of Interventions, Version 6 (updated July 2019); [Chapter 4]: Searching for the selected studies. *Cochrane* 2019

28. Page MJ, McKenzie JE, Bossuyt PM, et al. The PRISMA 2020 statement: an updated guideline for reporting systematic reviews. *BMJ* 2021;372:n71. doi: 10.1136/bmj.n71

For peer review only

For peer review only - http://bmjopen.bmj.com/site/about/guidelines.xhtml

FOOTNOTES

Acknowledgements

The protocol of the current umbrella review was registered in the PROSPERO database: CRD42020190700.

Availability of data and materials

The datasets used and/or analysed during the umbrella review will be available from the corresponding author on reasonable request.

Word Count

2,548 words.

For peer review only

For peer review only - http://bmjopen.bmj.com/site/about/guidelines.xhtml

Appendix 1: Search strategy for Ovid MEDLINE.

1. exp Decision Making/
2. exp Decision Support Techniques/
3. Decision Support Systems, Clinical/
4. "shared decision-making" OR "shared decision making" OR "decision process" OR "decision support" mp. [mp=title, abstract, original title, name of substance word, subject heading word, keyword heading word, protocol supplementary concept, rare disease supplementary concept, unique identifier]
5. ((process* OR support* OR aid* OR share* OR mak* OR individual* OR sharing OR informed) adj2 (decid* OR decision* OR choice)) mp. [mp=title, abstract, original title, name of substance word, subject heading word, keyword heading word, protocol supplementary concept, rare disease supplementary concept, unique identifier]
6. 1 OR 2 OR 3 OR 4 OR 5
7. exp Mental Disorders/
8. Mental Health/
9. Mentally Ill Persons/
10. ((mental* OR psychiatr* OR psycholog*) adj2 (problem* OR difficult* OR disorder* OR disease* OR ill* OR health*)) mp. [mp=title, abstract, original title, name of substance word, subject heading word, keyword heading word, protocol supplementary concept, rare disease supplementary concept, unique identifier]
11. 7 OR 8 OR 9 OR 10
12. exp Mood Disorders/ OR Depressive Disorder/ OR Bipolar Disorder/ OR affective disorder* OR depressive disorder* OR depression* OR mania* OR bipolar disorder* OR dysthymic disorder* OR dysthymia* OR affective disturbance* OR affective ill* OR mood disturbance*mp. [mp=title, abstract, original title, name of substance word, subject heading word, keyword heading word, protocol supplementary concept, rare disease supplementary concept, unique identifier]
13. exp Anxiety Disorders/ OR Neurotic Disorders/ OR Obsessive-Compulsive Disorder/ OR Panic Disorder/ OR Phobic Disorders/ OR Stress Disorders, Post-traumatic/ OR anxiety disorder* OR neurotic disorder* OR obsessive-compulsive disorder* OR panic disorder* OR phobic disorder* OR phobia* OR generalized anxiety disorder* OR generalised anxiety disorder* OR posttraumatic stress disorder* mp. [mp=title, abstract, original title, name of substance word, subject heading word, keyword heading word, protocol supplementary concept, rare disease supplementary concept, unique identifier]
14. exp "Trauma and Stressor Related Disorders"/ OR Stress Disorders, Traumatic/ OR Psychological Trauma/ OR Psychological Distress/ OR Stress, Psychological/ OR trauma* OR stress disorder* OR psychological distress* OR emotional distress* mp. [mp=title, abstract, original title, name of substance word, subject heading word, keyword heading word, protocol supplementary concept, rare disease supplementary concept, unique identifier]
15. exp Personality Disorders/ OR personality disorder* OR personality patholog* OR personality difficult* OR disordered personalit* mp. [mp=title, abstract, original title, name of substance word, subject heading word, keyword heading word, protocol supplementary concept, rare disease supplementary concept, unique identifier]
16. exp Substance-Related Disorders/ OR Alcohol-Related Disorders/ OR Illicit Drugs/ OR Alcoholism/ OR Binge Drinking/ OR "drug abuse" OR "substance abuse" OR "alcohol abuse" OR "drug dependence" OR "substance dependence" OR "alcohol dependence" OR "drug addiction" OR "substance addiction" OR "alcohol addiction" OR "substance-use disorder" OR "alcohol-use disorder" OR alcoholi* OR binge drink* mp. [mp=title, abstract, original title, name of substance word, subject heading word, keyword heading word, protocol supplementary concept, rare disease supplementary concept, unique identifier]

For peer review only

17. exp Affective Disorders, Psychotic/ OR Psychotic Disorders/ OR Paranoid Disorders/ OR Schizophrenia/ OR delusion* OR hallucinat* OR schizophren* OR "psychosis" OR "schizoaffective" OR "psychotic" OR "paranoid"
18. exp "Feeding and Eating Disorders"/
19. ((exp Anorexia Nervosa/ OR Anorexia/) OR (exp Bulimia Nervosa/ OR Bulimia/ OR Binge-Eating Disorder/)) OR ((anorexi* OR bulimi*) AND nervosa) OR eating disorder* OR binge-eat* OR (bing* adj2 eat*) OR (compulsive adj2 (eat* OR vomit* or purg*)) mp. [mp=title, abstract, original title, name of substance word, subject heading word, keyword heading word, protocol supplementary concept, rare disease supplementary concept, unique identifier]
20. 12 OR 13 OR 14 OR 15 OR 16 OR 17 OR 18 OR 19
21. ((systematic OR scoping OR literature) ADJ (review* OR overview*)) OR "review* of reviews" OR meta-analy* OR metaanaly* OR ((systematic OR evidence) ADJ1 assess*) OR metasynthe* OR meta-synthe*.tw. OR exp Review Literature as Topic/ OR exp Review/ OR Meta-Analysis as Topic/ OR Meta-Analysis/ OR "systematic review"/
22. 6 AND 11 AND 20 AND 21
23. Limit 22 to (English and yr= “2010-2021)

For peer review only

Reporting checklist for protocol of a systematic review and meta analysis.

Based on the PRISMA-P guidelines.

Instructions to authors

Complete this checklist by entering the page numbers from your manuscript where readers will find each of the items listed below.

Your article may not currently address all the items on the checklist. Please modify your text to include the missing information. If you are certain that an item does not apply, please write "n/a" and provide a short explanation.

Upload your completed checklist as an extra file when you submit to a journal.

In your methods section, say that you used the PRISMA-Preporting guidelines, and cite them as:

Moher D, Shamseer L, Clarke M, Ghersi D, Liberati A, Petticrew M, Shekelle P, Stewart LA. Preferred Reporting Items for Systematic Review and Meta-Analysis Protocols (PRISMA-P) 2015 statement. Syst Rev. 2015;4(1):1.

		Reporting Item	Page Number
**Title**			
Identification	#1a	Identify the report as a protocol of a systematic review	1, 2, 4
Update	#1b	If the protocol is for an update of a previous systematic review, identify as such	N/A
**Registration**			
	#2	If registered, provide the name of the registry (such as PROSPERO) and registration number	6, 15
**Authors**			
Contact	#3a	Provide name, institutional affiliation, e-mail address of all protocol authors; provide physical mailing address of corresponding author	1
Contribution	#3b	Describe contributions of protocol authors and identify the guarantor of the review	11

For peer review only

**Amendments**			
	#4	If the protocol represents an amendment of a previously completed or published protocol, identify as such and list changes; otherwise, state plan for documenting important protocol amendments	N/A
**Support**			
Sources	#5a	Indicate sources of financial or other support for the review	11
Sponsor	#5b	Provide name for the review funder and / or sponsor	11
Role of sponsor or funder	#5c	Describe roles of funder(s), sponsor(s), and / or institution(s), if any, in developing the protocol	N/A
**Introduction**			
Rationale	#6	Describe the rationale for the review in the context of what is already known	2, 4
Objectives	#7	Provide an explicit statement of the question(s) the review will address with reference to participants, interventions, comparators, and outcomes (PICO)	4, 5
**Methods**			
Eligibility criteria	#8	Specify the study characteristics (such as PICO, study design, setting, time frame) and report characteristics (such as years considered, language, publication status) to be used as criteria for eligibility for the review	6, 7, 8
Information sources	#9	Describe all intended information sources (such as electronic databases, contact with study authors, trial registers or other grey literature sources) with planned dates of coverage	8
Search strategy	#10	Present draft of search strategy to be used for at least one electronic database, including planned limits, such that it could be repeated	8
Study records - data management	#11a	Describe the mechanism(s) that will be used to manage records and data throughout the review	9
Study records - selection process	#11b	State the process that will be used for selecting studies (such as two independent reviewers) through each phase of the review (that is, screening, eligibility and inclusion in meta-analysis)	8

For peer review only

Study records - data collection process	#11c	Describe planned method of extracting data from reports (such as piloting forms, done independently, in duplicate), any processes for obtaining and confirming data from investigators	9
Data items	#12	List and define all variables for which data will be sought (such as PICO items, funding sources), any pre-planned data assumptions and simplifications	7, 8
Outcomes and prioritization	#13	List and define all outcomes for which data will be sought, including prioritization of main and additional outcomes, with rationale	8
Risk of bias in individual studies	#14	Describe anticipated methods for assessing risk of bias of individual studies, including whether this will be done at the outcome or study level, or both; state how this information will be used in data synthesis	9
Data synthesis	#15a	Describe criteria under which study data will be quantitatively synthesised	10
Data synthesis	#15b	If data are appropriate for quantitative synthesis, describe planned summary measures, methods of handling data and methods of combining data from studies, including any planned exploration of consistency (such as I2, Kendall's τ)	10
Data synthesis	#15c	Describe any proposed additional analyses (such as sensitivity or subgroup analyses, meta-regression)	10
Data synthesis	#15d	If quantitative synthesis is not appropriate, describe the type of summary planned	N/A
Meta-bias(es)	#16	Specify any planned assessment of meta-bias(es) (such as publication bias across studies, selective reporting within studies)	9
Confidence in cumulative evidence	#17	Describe how the strength of the body of evidence will be assessed (such as GRADE)	9,10

The PRISMA-P elaboration and explanation paper is distributed under the terms of the Creative Commons Attribution License CC-BY. This checklist was completed on 14. March 2021 using https://www.goodreports.org/, a tool made by the EQUATOR Network in collaboration with Penelope.ai

For peer review only
